# Peritonsillar Phlegmon: An Addition to the Spectrum of COVID-19

**DOI:** 10.7759/cureus.12369

**Published:** 2020-12-29

**Authors:** Muhammad Atique Alam Khan, Nathaniel Rosal, Iqra Iqbal, Artem Minalyan

**Affiliations:** 1 Internal Medicine, Abington - Jefferson Health, Abington, USA

**Keywords:** peritonsillar phlegmon, covid-19, tonsillitis, lymphadenopathy

## Abstract

The coronavirus disease 2019 (COVID-19) epidemic, caused by severe acute respiratory syndrome coronavirus 2 (SARS-CoV-2), was first reported in December 2019 in Wuhan, China, then declared to be a pandemic associated with substantial morbidity and mortality. It has shown to exhibit a vast array of symptoms, among which fever, shortness of breath, and cough are the most commonly reported. Lymphadenopathy and tonsillar enlargement is a less common finding reported with this infection. This case describes a patient with tonsillar inflammation which was complicated by peritonsillar phlegmon, with negative throat culture and positive COVID-19 test, suggesting a COVID-19-related etiology of the disease. After the literature search, to the best of our knowledge, this is the first reported case of COVID-related peritonsillar inflammation and phlegmon formation.

## Introduction

In the months following the emergence of the coronavirus disease 2019 (COVID-19) pandemic, many different clinical findings were reported in the literature. Most of the published literature to date focuses upon the severe respiratory presentation of COVID-19 such as pneumonia and acute respiratory distress syndrome (ARDS). Some cases have been reported to have acute thromboembolic events such as deep venous thrombosis and pulmonary embolism. Although a majority of these patients have been found to report a combination of nonspecific viral symptoms, such as fever, myalgia, arthralgia, some cases have presented as sore throat as well. We did a literature search for patients who presented with a sore throat complicated by tonsillitis and peritonsillar abscess but did not find any other cases of COVID-19-related peritonsillar abscess. Hence, we report a case of peritonsillar phlegmon and head and neck lymphadenopathy in a patient diagnosed with COVID-19.

## Case presentation

**Figure 1:**
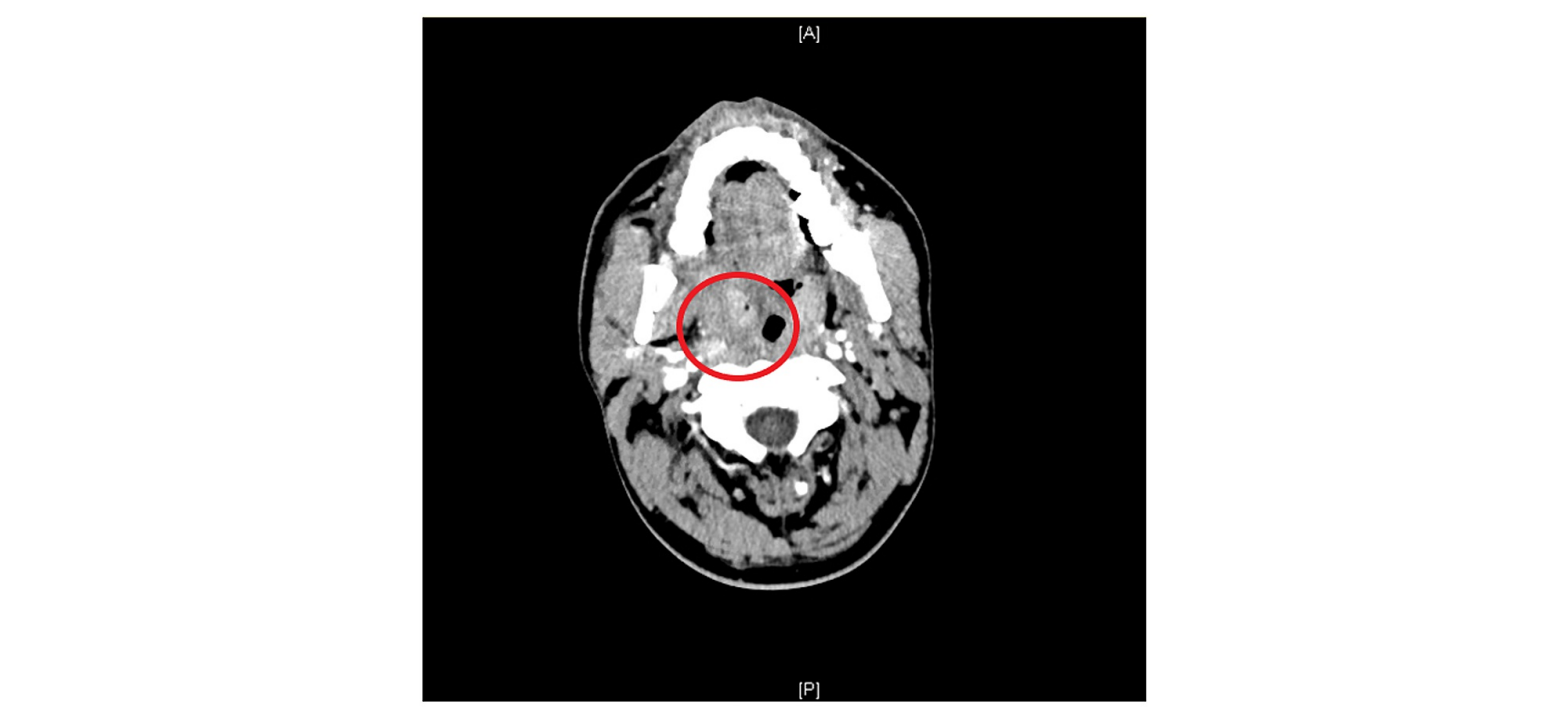
Right tonsillar enlargement and peritonsillar tracking

A 33-year-old male with no significant past medical history presented to the emergency room with a chief complaint of a sore throat. He noted the symptoms began one week prior and were associated with dysphagia, decreased oral intake, and intermittent fevers. He denied symptoms of shortness of breath, chest pain, myalgias, joint pains, nausea, vomiting, or diarrhea. Vital signs upon admission revealed a temperature of 98.4 degrees F, blood pressure of 135/65 mmHg, heart rate of 81 beats per minute, a respiratory rate of 20 breaths per minute, and an oxygen saturation of 99% on ambient air. Physical exam upon admission revealed a comfortable, awake, alert, and oriented patient. Cardiac examination revealed a regular rate and rhythm with normal S1 and S2 and no murmurs. The pulmonary exam revealed clear lungs to anterior and posterior auscultation with no wheezes, rales, or diminished breath sounds. Abdominal exam revealed normal bowel sounds, and a soft, non-tender, non-distended abdomen. Oral and neck exam was significant for an erythematous and swollen right tonsil and tenderness to palpation of the right anterior neck with associated submandibular and supraclavicular lymphadenopathy, respectively. Laboratory findings were significant for a leukocytosis to 16.5 K/UL. A CT scan of the head and neck was obtained and was significant for right peritonsillar edema with tracking along the lateral pharyngeal wall consistent with phlegmon (Figure 1).

Associated lymphadenopathy included the involvement of the internal jugular chain, posterior triangle, bilateral supraclavicular, and submandibular lymph nodes. The epiglottis was without enlargement. The paranasal sinuses and mastoid air cells were clear. A throat swab was obtained for bacterial culture, and meanwhile, clindamycin was started. Blood cultures were obtained too. When the cultures came back negative, the empiric antibiotic was stopped. A monospot was obtained which was negative. A nasopharyngeal swab for COVID-19 was positive for SARS-CoV-2 ribonucleic acid (RNA). The patient was discharged after one day in the hospital and was instructed to remain in quarantine. Upon telemedicine follow-up one week later, the patient reported marked improvement in dysphagia and denied fevers after symptomatic treatment and rest.

## Discussion

About 50-80% of cases of pharyngitis are secondary to viral infection. The most common viruses are rhinovirus, influenza, adenovirus, coronavirus, and parainfluenza. The most common bacterial infection is Group A beta-hemolytic streptococci, which causes 5% to 36% of cases of acute pharyngitis. Other bacterial etiologies include Group B & C streptococci, Chlamydia and Mycoplasma pneumonia, Haemophilus influenzae, Neisseria meningitides, Neisseria gonorrhoeae, Fusobacterium necrophorum, and Corynebacterium diphtheriae [1]. Coronaviruses are nonsegmented enveloped RNA viruses with a single-strand linear positive-sense RNA. Six types of coronavirus have been identified that cause human disease. Four of these cause mild respiratory symptoms whereas the other two, Middle East respiratory syndrome (MERS-CoV) coronavirus and severe acute respiratory syndrome coronavirus (SARS-CoV-1), have previously resulted in epidemics with high mortality rates. [2]. In December 2019, SARS-Cov-2 infection started to emerge in the city of Wuhan, China. Since that moment, COVID-19 has dramatically spread all over the world crossing all countries’ borders until the World Health Organization (WHO) confirmed it as a pandemic disease on March 11, 2020.

According to an analysis done by Shi et al., out of 81 patients admitted with COVID-19 in Wuhan, only 6% of patients had lymphadenopathy. There was a wide array of radiographic findings noted in patients with COVID-19 infection. The commonest patterns noted were bilateral pulmonary interstitial ground-glass opacities. Other findings that were less commonly reported were interlobular septal thickening, air bronchogram, and pleural effusion, but lymph node enlargement was not commonly seen [3]. Contrary to this study in China, Valette et al. described an analysis performed in critically ill patients in France, where some patients did have significant lymphadenopathies, especially in the mediastinum [4]. A small number of COVID-19 cases have also been reported with parotitis and associated cervical lymphadenopathy. Ear nose throat (ENT) specialists have reported an increased incidence of ear nose and throat symptoms during the pandemic. The loss of sensation of taste and smell also points towards the locoregional effects of the virus in the head and neck area [5].

In a large database analysis done by El-Anwar et al., 1773 patients were reviewed who had tested positive for COVID-19. The purpose of the analysis was to describe the ENT symptoms in COVID-19 affected patients. Although these symptoms are not as common as fever and cough, they are still present in many patients and need to be mentioned. The analysis found that the most common ENT manifestations of COVID-19 were sore throat (11.3%) and headache (10.7%). Other manifestations were pharyngeal erythema (5.3%), nasal congestion (4.1%), rhinorrhea (2.1%), upper respiratory tract infection (URTI) (1.9%), and tonsil enlargement (1.3%) [6]. In this large analysis, we do see that a few cases have been associated with tonsillar enlargement, but a complicated peritonsillar phlegmon has not been reported before.

In our case, the throat culture was negative, decreasing the possibility of a bacterial sore throat, while the COVID-19 test was positive, making the probability of COVID-19-related peritonsillar phlegmon more likely. As SARS-CoV-2 continues to run rampant in most countries, this case presents a previously unreported constellation of symptoms and adds to the growing body of literature regarding the spectrum of this novel coronavirus. 

## Conclusions

Our case report points out an important and unique clinical finding seen with COVID-19 infection. It emphasizes that we should stay vigilant about the ENT-related findings of COVID-19, including tonsillitis and peritonsillar inflammation. This condition can cause a threat to the airway in serious conditions. These patients may develop a need for intubation and antibiotics for the airway compromise and superimposed bacterial infection, respectively. Since the data is still emerging on the pathologic spectrum of COVID-19 infection, more studies need to be done to document any cases of lymphadenopathy and peritonsillar inflammation that are thought to be related to COVID-19. Throat cultures should also be obtained to make sure that symptoms are not caused by a bacterial infection in the very first place, mandating the earlier use of antibiotics in that situation.
